# Intratumoral heterogeneity of *EGFR*-activating mutations in advanced NSCLC patients at the single-cell level

**DOI:** 10.1186/s12885-019-5555-y

**Published:** 2019-04-23

**Authors:** Longhua Guo, Zhihong Chen, Chongrui Xu, Xuchao Zhang, Honghong Yan, Jian Su, Jinji Yang, Zhi Xie, Weibang Guo, Feng Li, Yilong Wu, Qing Zhou

**Affiliations:** 1 Guangdong Lung Cancer Institute, Guangdong Provincial Key Laboratory of Translational Medicine in Lung Cancer, Guangdong Provincial People’s Hospital and Guangdong Academy of Medical Sciences, 106 Zhongshan Er Road, Guangzhou, 510080 People’s Republic of China; 2 Department of Medical Oncology, Cancer Center, Meizhou People’s Hospital (Huangtang Hospital), Meizhou Hospital Affiliated to Sun Yat-Sen University, 63 Huangtang Road, Meizhou, 514031 People’s Republic of China

**Keywords:** Intratumoral heterogeneity, Single-cell analysis, *EGFR*-activating mutations

## Abstract

**Background:**

Intratumoral epidermal growth factor receptor (*EGFR*) mutational heterogeneity is yet controversial in non-small cell lung cancer (NSCLC) patients. Single-cell analysis provides the genetic profile of single cancer cells and an in-depth understanding of the heterogeneity of a tumor.

**Methods:**

Firstly, single H1975 cells harboring the *EGFR* L858R mutation were submitted to flow cytometry isolation, nested polymerase chain reaction (nested-PCR) amplification, and direct DNA sequencing to assess the feasibility of single-cell direct DNA sequencing. Then, the single cells of patients with lung adenocarcinoma receiving gefitinib were captured by laser capture microdissection and analyzed by the above methods to identify the intratumoral heterogeneity of the *EGFR* L858R mutant. Three patients with progression-free survival (PFS) > 14 months were categorized as the long PFS group, and 3 patients with PFS < 6 months as the short PFS group. The correlation between the abundance of *EGFR* L858R mutant and PFS was analyzed.

**Results:**

104 single H1975 cells were isolated. 100/104 were amplified by nested-PCR and confirmed by direct sequencing. We captured 135 tumor cells from the tissues of six patients. 120 single tumor cells were successfully amplified and sequenced. The rate of *EGFR* exon 21 mutation was only 77.5% (93/120). Furthermore, the rate of mutation in exon 21 of *EGFR* was significantly higher in the long PFS group than in the short PFS group (86.4 ± 4.9% vs. 68.9 ± 2.8%, *P* = 0.021).

**Conclusion:**

Our study suggested the intratumoral heterogeneity of *EGFR*-activating mutations in lung adenocarcinoma confirmed on the single-cell level, which might be associated with EGFR-TKIs response in lung adenocarcinoma patients harboring the *EGFR* L858R mutation.

**Electronic supplementary material:**

The online version of this article (10.1186/s12885-019-5555-y) contains supplementary material, which is available to authorized users.

## Background

Lung cancer is a leading cause of cancer-related deaths all over the world [1]. In recent years, several large randomized controlled clinical trials consistently demonstrated that the tyrosine kinase inhibitors (TKIs) of the epidermal growth factor receptor (EGFR) have a great efficacy in the treatment of patients with non-small cell lung cancer (NSCLC) harboring an *EGFR-*activating mutation compared to chemotherapy as the first-line treatment [2–10]. The response to EGFR-TKIs is different in patients carrying mutant *EGFR*. Some patients experienced a progression-free survival (PFS) of > 1 year, whereas others presented a PFS of < 6 months. Recently, intratumoral heterogeneity, intertumoral heterogeneity, and pre- or post-treatment heterogeneity regarding *EGFR*-activating mutations have been considered as potential causes for the differences in response to EGFR-TKIs [11–14].

Intratumoral heterogeneity arises from the introduction of genetic and epigenetic alterations by genomic and chromosomal instability and different patterns of clonal evolution over space and time, and has been reported in various types of cancers, including lung cancer [15, 16]. Nevertheless, studies on the intratumoral heterogeneity of *EGFR*-activating mutations generated contradicting results [17–19]. These discrepancies might be attributed to the different samples processing methods, including multi-region bulk tissue sampling and manual microdissection analysis. Indeed, these two methods allow the examination on the tissue or multi-cell levels [20]. Consequently, the intrinsic cell-to-cell variation in the *EGFR* mutation status might be masked by bulk-cell examination and the mutation status of *EGFR* might be misinterpreted due to the interference from the genetic heterogeneity of the cancer cells. Single-cell analysis directly provides the genetic status of single cancer cells and an in-depth understanding of the genetic characteristics of a tumor by isolating the single cells by flow cytometry (FCM) and laser capture microdissection (LCM) [21]. In addition, single tumor cell analysis might provide a deeper insight into the occurrence of intratumoral heterogeneity of EGFR-activating mutations [22].

In the present study, we investigated the intratumor heterogeneity with single-cell analysis as a definitive approach. Single H1975 cells that harbor the *EGFR* L858R heterozygous mutation in exon 21 were isolated by FCM and used for evaluating the feasibility of single-cell analysis of the mutation. A previous study by our group demonstrated the presence of *EGFR* heterogeneity on the tissue level and showed that the relative abundance of *EGFR* mutation in tumor tissues could predict the benefit of EGFR-TKI treatments [23]. Based on the single-cell method, we explored whether *EGFR* activating mutation heterogeneity in a tumor did exist in actual lung adenocarcinoma specimens positive for the L858R mutation in exon 21 of *EGFR* and its relation to EGFR-TKI response.

## Methods

### Cell culture, single cell isolation, and DNA extraction

The NSCLC cell line H1975, which harbors the L858R heterozygous mutation in exon 21 of the *EGFR* gene [24], was a kind gift from Professor Tony S. Mok (Prince of Wales Hospital, Hong Kong), and was originally purchased from the American Type Culture Collection (ATCC). The H1975 cells were cultured in RPMI 1640 containing 10% fetal calf serum and incubated at 37 °C in a humidified atmosphere with 5% CO_2_. When the cells achieved 80–90% confluency, they were trypsinized to prepare single-cell suspensions that were seeded in 96-well plates and lysed with 10 μL cell lysis solution (50 mmol/L Tris, 1 mmol/L EDTA, 0.5% Tween-20, and 200 mg/L proteinase K). The single cells were isolated using a FASCArial II system (BD Biosciences, Franklin Lake, NJ, USA). Before our study, we had conducted a preliminary experiment for single cell isolation with the FASCArial II system. H1975 suspension was labelled with Trypan and single cell was sorted by the FCM onto a microscope slide. Then we found the droplet on the slide and confirm whether there was a single cell in it under the microscope (Additional file 1: Figure S1). As previously reported, the rate of successful single cell isolation was > 95% and the present study used the optimized parameters (e.g., Ampl:16.4; Drop 1:440; Gap:12; Drop Delay:28.26) from the preliminary experiment [25].

### Patients and tissue samples

**Fig. 1:**
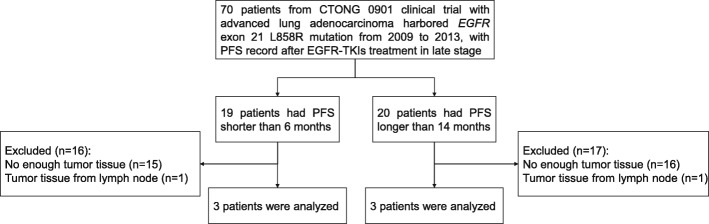
Study flowchart

Patients from the CTONG 0901 clinical trial, treated at the Guangdong Provincial People's Hospital from 2009 to 2013, were screened (Fig. 1) [26]. The inclusion criteria were: 1) first-line treatment with gefitinib; 2) available PFS data; 3) adequate surgical and frozen specimens of > 1 cm in diameter and preserved at the tumor tissue biobank of the Guangdong Lung Cancer Institute (GLCI) [No. GDREC2013185H(R2)]; 4) pure adenocarcinoma; and 5) *EGFR* L858R mutation in exon 21 by direct sequencing. In order to evaluate the effect of the *EGFR* mutation on patient prognosis, three patients with PFS > 14 months (long PFS) and three patients with PFS < 6 months (short PFS) were included (Fig. 1). Tissues from all eligible patients were subjected to LCM and single-cell analysis.

### Single cell isolation by LCM and DNA extraction from tissue samples

**Fig. 2:**
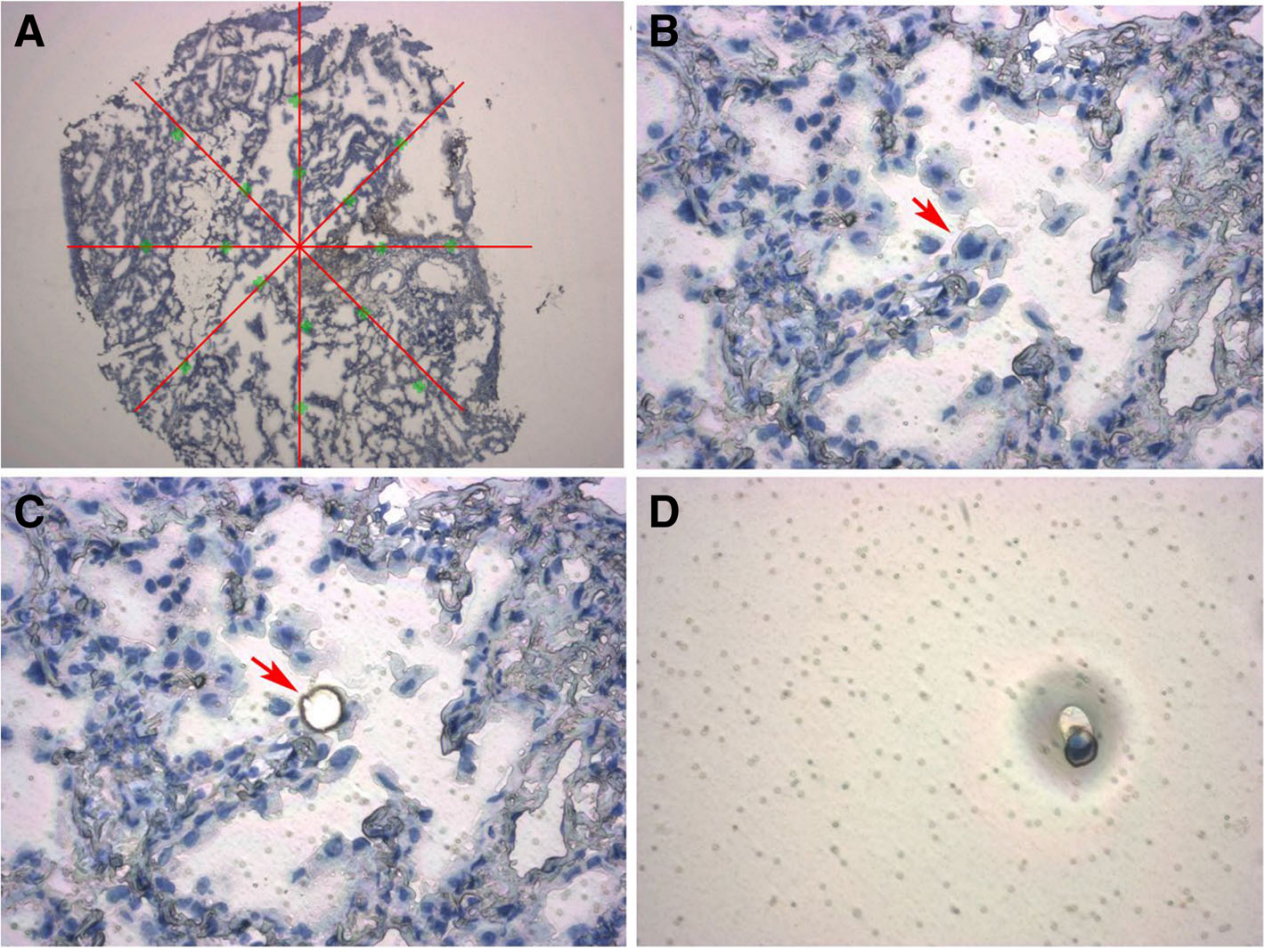
Single tumor cell isolation by laser capture microdissection (LCM). **a** Single tumor cells capture map. The green crosses represent the sites of single tumor cells captured along six evenly spaced lines radiating from the center of the tumor section (with neighboring sites located approximately 2 mm apart on the lines). In each sample, 20–24 tumor cells were captured (1 cell/site). **b**, **c**, **d** Example of a single cell being sampled from a 5-μm section using the Arcturus system. A target cell (red arrow) was identified on the microdissection system, dissected with an ultraviolet laser, and collected by adhesion onto an infrared laser melted polymer film. **b** Whole section before sampling. **c** Whole section after sampling the single cell. **d** The sampled single cell

For each frozen tissue sample, a 5-μm-thick section was mounted on a membrane slide (Arcturus; Thermo Fisher Scientific, Waltham, MA, USA) and stained with hematoxylin for the histomorphological identification of the cells. Dehydration was performed with 70% ethanol for 30 s, 95% ethanol for 30 s, and 100% ethanol for 30 s. Then, the slides were placed in xylene for 5 min. After air-drying, the sections were microdissected to capture individual tumor cells using the LCM system (Arcturus) according to the manufacturer’s protocol. In each section, 20–24 points were selected and one single tumor cell was captured at each point. Figure 2 shows the cell capture map and an example of a section being sampled. According to microscopy, 12 normal cells on the periphery of the tumors were captured as negative controls. In the Arcturus system, the ultraviolet laser cuts the required tumor cells, whereas the infrared laser melts the thermolabile polymers for cell capture. Following microdissection, the cell lysis solution was directly added to the polymer film and placed into a 500-μL microcentrifuge tube [27].

### Single-cell nested polymerase chain reaction (nested-PCR) analysis

Whole DNA extracted from a single cell was subjected to nested-PCR amplification of the *EGFR* 21 exon. The primers and nested-PCR protocol are shown in Table 1 [28]. Briefly, for external PCR amplification, the 25-μL reaction volume contained 10 μL of single cell lysate, 0.5 μl of primers (Takara Bio, Otsu, Japan), and 1× GoTaq Colorless Master Mix (Promega, Madison, WI, USA). For internal PCR amplification, the 25-μL reaction volume consisted of 0.1 μL of the product of the external PCR amplification, 10 μL RNA-free water, 0.5 μL of internal primers, and 1× GoTaq Colorless Master Mix. The single-cell nested PCR products were visualized by 2% agarose gel electrophoresis. Moreover, for the single cell nested-PCR of the H1975 cell line, two blank controls (no cells were isolated) were used for every six H1975 cells. Among the cancer cells captured from the tissues, DNA extracted from cancerous tissues for nested-PCR amplification was used as positive control and that from two normal cells from each section was used as negative controls.

**Table 1:** Nested-PCR amplification primers and protocols for *EGFR* exon 21

	Primers	Sequence 5′-3′	PCR conditions	PCR product (bp)
External PCR amplification	External	TCAGAGCCTGGCA	1) 95 °C, 5 min	297
	Forward	TGAACATGACCCTG	61 °C, 30 s 72 °C, 30 s 3) 72 °C, 7 min2)35 cycles:	
			95 °C, 45 s	
	External	GGTCCCTGGTGTC	61°C, 30 s	
	Reverse	AGGAAAATGCTGG	72°C, 30 s 3) 72°C, 7 min	
Internal PCR amplification	Internal	ATGAACTACTTG	1) 95 °C, 5 min	188
	Forward	GAGGACCGT	2)35 cycles:	
	Internal	GAAAATGCTGGCT	95 °C, 45 s	
	Reverse	GACCTAAG	60 °C, 30 s 72 °C, 30 s 3)72 °C, 7 min	

### *EGFR* mutation analysis by direct sequencing

All nested-PCR amplified products based on single cells that showed positive PCR reactions by agarose gel electrophoresis were sequenced to determine the *EGFR* exon 21 status. The products were purified, labeled using the BigDye Terminator v3.1 Cycle Sequencing Kit (Applied Biosystems, Foster City, CA, USA), and sequenced using an ABI 3100 Genetic Analyzer (Applied Biosystems). The sequence reactions were confirmed by two experienced independent readers (Xuchao Zhang and Zhihong Chen).

### Statistical analysis

For the H1975 cell line, the efficiency of nested-PCR amplification and the rate of allele drop-out (ADO) were assessed by direct sequencing. For the tumor specimens, the efficiency of nested-PCR and the mutational rate in each sample were computed first. Subsequently, PCR efficiency and the mutation rate between the two groups were analyzed and compared by the chi-square test. Statistical analysis was carried out using SPSS 21.0 (IBM, Armonk, NY, USA). Two-sided *P* < 0.05 was considered statistically significant.

## Results

### Validation of the heterozygous mutation of *EGFR* L858R in H1975 cells

**Fig. 3:**
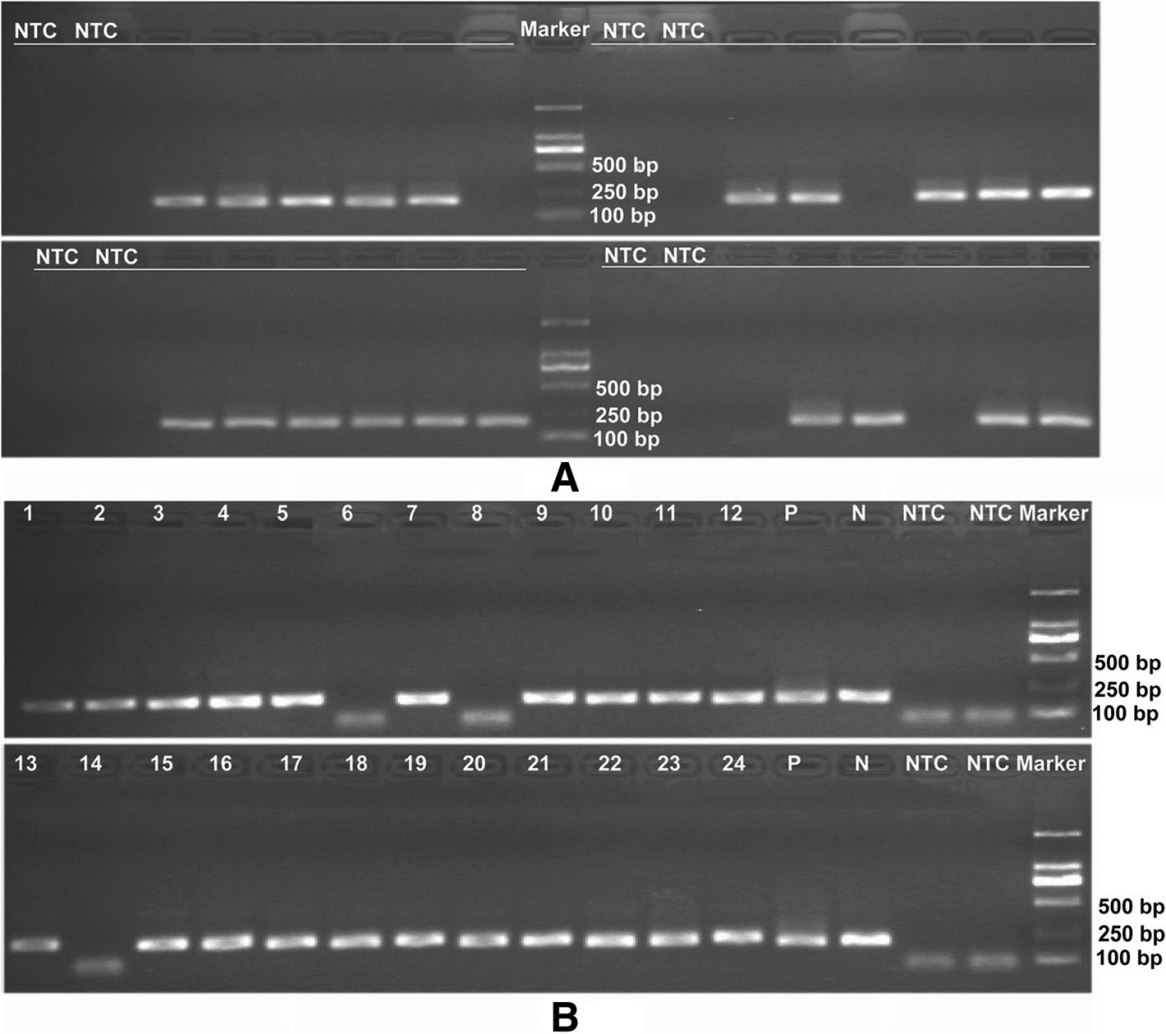
Representative agarose gel electrophoresis pictures from single-cell nested PCR amplification. **a** Agarose gel electrophoresis of single H1975 cells. **b** Agarose gel electrophoresis of single tumor cells. (P: tissue cells as positive control; N: normal cell as negative control; NTC: blank control)

**Fig. 4:**
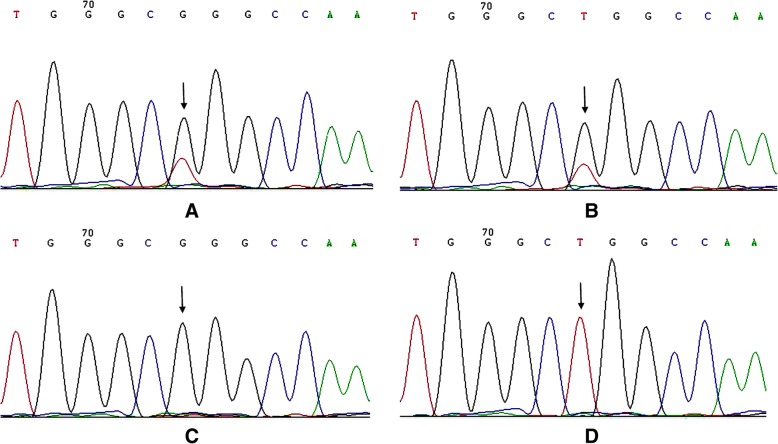
Representative sequencing picture for *EGFR* exon 21 from single H1975 cells. **a** Heterozygous mutant sequence for single a H1975 cell. **b** Another heterozygous mutant sequence for single a H1975 cell. **c** Homozygous mutant sequence for a single H1975 cell. **d** Homozygous wild type sequence for a single H1975 cell

Feasibility was tested using single H1975 cells to validate the single-cell analysis of *EGFR* mutation in NSCLC cells. A total of 104 individual H1975 cells were isolated by FCM. The success rate of nested-PCR for *EGFR* 21 exon amplification in single H1975 cells was 96.2% (100/104). Figure 3a shows a representative image of agarose gel electrophoresis from nested-PCR amplification of single H1975 cells. All samples positive for *EGFR* 21 exon amplification yielded sufficient DNA for successful sequencing. The DNA sequencing results showed that the *EGFR* L858R heterozygous mutation was detected in 93% of the cells (93/100) (Fig. 4a-b). The homozygous mutation and wild-type alleles were detected in four and three cells, respectively, indicating that ADO putatively occurred during the nested-PCR process (Fig. 4c-d). The rate of ADO was 7.0% (7/100).

### Intratumoral heterogeneity of *EGFR* mutation in lung adenocarcinoma tissues

After confirming the validity of the single-cell analysis system, we examined the presence of intratumoral heterogeneity of the *EGFR* mutation in adenocarcinoma NSCLC tumor tissues. In total, 135 tumor cells and 12 normal cells as negative controls were captured by LCM from the tissue samples obtained from six patients. The *EGFR* exon 21 was successfully amplified by nested-PCR in 120 tumor cells, including 61 cells from the short PFS group and 59 cells from the long PFS group (Additional file 2: Table S2). A representative agarose gel electrophoresis image of single tumor cells from tissue resections is shown in Fig. 3b. For the 12 negative controls from single normal cells, 10 normal cells were amplified successfully by nested-PCR for *EGFR* exon 21.

Among the 120 single tumor cells amplified successfully, DNA sequencing showed that the overall rates of *EGFR* L858R mutation and wild type gene were 77.5% (93/120) and 22.5% (27/120), respectively. None of the six samples presented a 100% mutation rate, thereby confirming the presence of intratumoral heterogeneity.

### Association between intratumoral *EGFR* mutation rate and patient prognosis

To evaluate the effect of intratumoral *EGFR* L858R mutation rate on the patients’ response to gefitinib therapy, we screened for patients with short (< 6 months) and long (> 14 months) PFS for analysis. The PFS of the long PFS group were 15, 19, and 22 months, while those of the short PFS group were 1, 3, and 5 months. The baseline characteristics of the six subjects are shown in Additional file 2: Table S1.

The data showed that the mutation abundance of *EGFR* L858R was higher in the long PFS group than in the short PFS group (86.4 ± 4.9% vs. 68.9 ± 2.8%, *P* = 0.021) and the absolute difference was 17.5% (Table 2).

**Table 2:** Rates of *EGFR* exon 21 mutation

Patient no.	Cells number	Mutation rate	*P*-value
Long PFS group			0.021
No.1	19	17 (89.5%)	
No.2	21	17 (81.0%)	
No.3	19	17 (89.5%)	
Total	59	51 (86.4%)	
Short PFS group			
No.4	18	13 (72.2%)	
No.5	21	14 (66.7%)	
No.6	22	15 (68.2%)	
Total	61	42 (68.9%)	

## Discussion

Single-cell analysis directly provides the genetic status of single cancer cells and an in-depth understanding of the genetic heterogeneity of the tumor. Therefore, this study evaluated the feasibility of single-cell analysis of *EGFR* L858R mutation using H1975 cells, to detect the presence of intratumoral heterogeneity in lung adenocarcinoma. The single H1975 cells showed that the *EGFR* L858R mutation could be reliably detected using single-cell analysis. Herein, we reported for the first time the presence of intratumoral heterogeneity of the *EGFR* L858R mutation in lung adenocarcinoma on a single-cell level. Indeed, *EGFR* wild type tumor cells were detected in all six tumor samples. Furthermore, we presented preliminary evidence that the intratumoral *EGFR* L858R heterogeneity, as measured by the mutation rate, might be associated with the patients’ response to gefitinib therapy.

In the present study, we validated the feasibility of a single-cell analysis system for the detection of *EGFR* L858R mutation in H1975 cells, using a previously described method [21]. Our study demonstrated a high amplification rate (96.2%) of the *EGFR* exon 21 by nested-PCR, similar to that described previously [28]. During single-cell analysis, ADO is a stochastic and unique issue pertaining single-cell PCR that can alter the results. In ADO, only one of the two alleles present in a cell is amplified and detected after PCR; therefore, a heterozygous cell will appear homozygous [29]. The present study suggested that ADO led to a 7% error rate in the detection of *EGFR* mutation in exon 21, which was within the 5–15% range observed in some reports in the field of preimplantation genetic diagnosis (PGD) [29, 30]. In the present study, the contamination of the single cell could also have been an issue. Blank controls were used for every six single cells tested, and non-specific amplification was not detected. This strongly suggests that the procedure for nested-PCR amplification did not introduce any DNA contamination.

According to the results of single-cell analysis, *EGFR* wild type tumor cells were detected in all six tumor samples, which suggested the existence of intratumoral heterogeneity for *EGFR* mutation in lung adenocarcinoma. These results were in agreement with those from previous studies [11, 12, 14, 23]. Nevertheless, Yatabe et al. reported that the intratumoral heterogeneity of *EGFR* mutation was rare in lung adenocarcinoma [17]. The authors regarded the detected intratumoral *EGFR* mutation heterogeneity as pseudo-heterogeneity resulting from the mutant allele-specific imbalance (MASI) [24, 31]. In some areas within a tumor, *EGFR* can be mutated but not amplified. In the absence of the normal cells in these areas, the mutation signal would be equivalent to that of the wild-type; however, a tumor is always mixed with normal cells, which might result in a diluted *EGFR* mutation signal that is below the detection threshold of the method. Therefore, pseudo-heterogeneous distribution of *EGFR* mutation is observed in lung cancer. In this study, the single-cell approach allowed the analysis of tumor cells without the interference of normal cells, and pseudo-heterogeneity was not observed. Recently, Gerlinger et al. also demonstrated the presence of marked intratumoral heterogeneity with respect to somatic mutations in driver and passenger genes [32]. Taken together, these results strongly suggest that the *EGFR*-activating mutation status is heterogeneous in lung adenocarcinoma.

Several large randomized controlled clinical trials (e.g., IPASS [2], WJTOG 3405 [3], OPTIMAL [4], EURTAC [5], LUX-Lung 3 [6], First-SIGNAL [8], NEJ002 [9], LUX-Lung 6 [10]) consistently demonstrated that patients with NSCLC harboring the EGFR-activating mutation experienced a 9–13-month median PFS when they received EGFR-TKIs treatment. Therefore, we defined PFS > 14 months as long PFS. To evaluate the effect of intratumoral heterogeneity on patient prognosis, we screened for patients with PFS that was representative of short (< 6 months) and long (> 14 months) PFS. The present study suggested that the abundance of the *EGFR* L858R mutation in the long PFS group was higher than in the short PFS group (86.4 ± 4.9% vs. 68.9 ± 2.8%). The results indicated that intratumoral heterogeneity of *EGFR*-activating mutation might affect the benefits from EGFR-TKIs treatment. During EGFR-TKIs treatment, the *EGFR* mutant tumor cells are inhibited, whereas the *EGFR* wild-type tumor cells continue to proliferate. Thus, the high *EGFR* mutation heterogeneity in tumors could affect the response to EGFR-TKIs. These results are in agreement with those from previous studies [11, 12, 14], including one by our group, which showed that the relative abundance of the *EGFR* mutation in tumor tissues was associated with the benefits from EGFR-TKIs treatment [23]. Notably, as opposed to the pooled relative abundance measured at the tissue levels by previous studies, single-cell analysis allowed the direct calculation of intratumoral *EGFR* mutation rates, which might be sensitive indices for patient response to EGFR-TKIs therapy. Nevertheless, the number of patients was quite small and there are many factors influencing the effectiveness of EGFR-TKIs and PFS, including environmental and genetic factors. Therefore, the association between intratumoral heterogeneity of *EGFR* mutation and response to EGFR-TKIs therapy needs further research.

The PFS was used as the selection criterion for the patients, followed by detection of the heterogeneity of *EGFR*. Therefore, the reporting and detection bias were small. Nevertheless, the present study has some limitations. Firstly, only one exon of *EGFR* was tested owing to the small amount of DNA in a single cell. According to the previous reports on the application of whole genome amplification (WGA), sufficient DNA can be obtained to determine the *EGFR* status at multiple sites [33]. Secondly, the issue of ADO is inevitable in the process of single-cell PCR amplification, which might lead to false-negative results. Although the frequency of ADO was low in the detection of *EGFR* L858R mutation, it should be considered for the precision of the test; fluorescence PCR or digital PCR can be used to reduce the occurrence of ADO [33]. Finally, the relationship between intratumoral heterogeneity and EGFR-TKIs efficacy should be substantiated using large-sized sample studies and other methods like heterozygozity of the mutation, copy numbers, and immunohistochemistry(IHC). Despite these limitations, the present study provides some evidence for the existence of intratumoral heterogeneity of *EGFR*-activating mutation in lung adenocarcinoma at the level of single cells.

## Conclusion

In conclusion, the present study confirmed the intratumoral heterogeneity of EGFR-activating mutation in lung adenocarcinoma by single-cell analysis. The intratumoral heterogeneity of the *EGFR* L858R mutation might be associated with the gefitinib response in patients with adenocarcinoma NSCLC harboring the mutation. Further studies are needed to explore the potential factors influencing response to EGFR-TKIs in lung adenocarcinoma with *EGFR* activating mutant.

## Additional files

Additional file 1: **Figure S1.** Preliminary experiment for single cell isolation with flow cytometry (FCM). (A) Forward scattered light area (FSC-A) represents the cell size and side scattered light area (SSC-A) shows the number of cells. The red region represents the population of FCM collection. (B) H1975 cells in suspension were stained with Trypan blue and the single cells were sorted by FCM onto a microscope slide. The single cell (arrow) inside the droplet was observed under a microscope. (TIF 3317 kb)

Additional file 2: **Table S1.** Clinicopathological features of six patients with non-small cell lung cancer. **Table S2.** Efficiencies of nested PCR. (DOCX 24 kb)

## Abbreviations

ADO: Allele drop-out
ATCC: American Type Culture Collection
*EGFR*: epidermal growth factor receptor
FCM: Flow cytometry
GLCI: Guangdong Lung Cancer Institute
IHC: Immunohistochemistry
LCM: Laser capture microdissection
NSCLC: Non-small cell lung cancer
PCR: Polymerase Chain Reaction
PFS: progression-free survival
PGD: Preimplantation genetic diagnosis
TKIs: Tyrosine kinase inhibitors
WGA: Whole genome amplification
