# Supplementary figures and images for: Intratumoral heterogeneity of EGFR-activating mutations in advanced NSCLC patients at the single-cell level
Source: BMC Cancer. 2019 Apr 23;19:369. doi: 10.1186/s12885-019-5555-y (PMC6480785; doi:10.1186/s12885-019-5555-y)

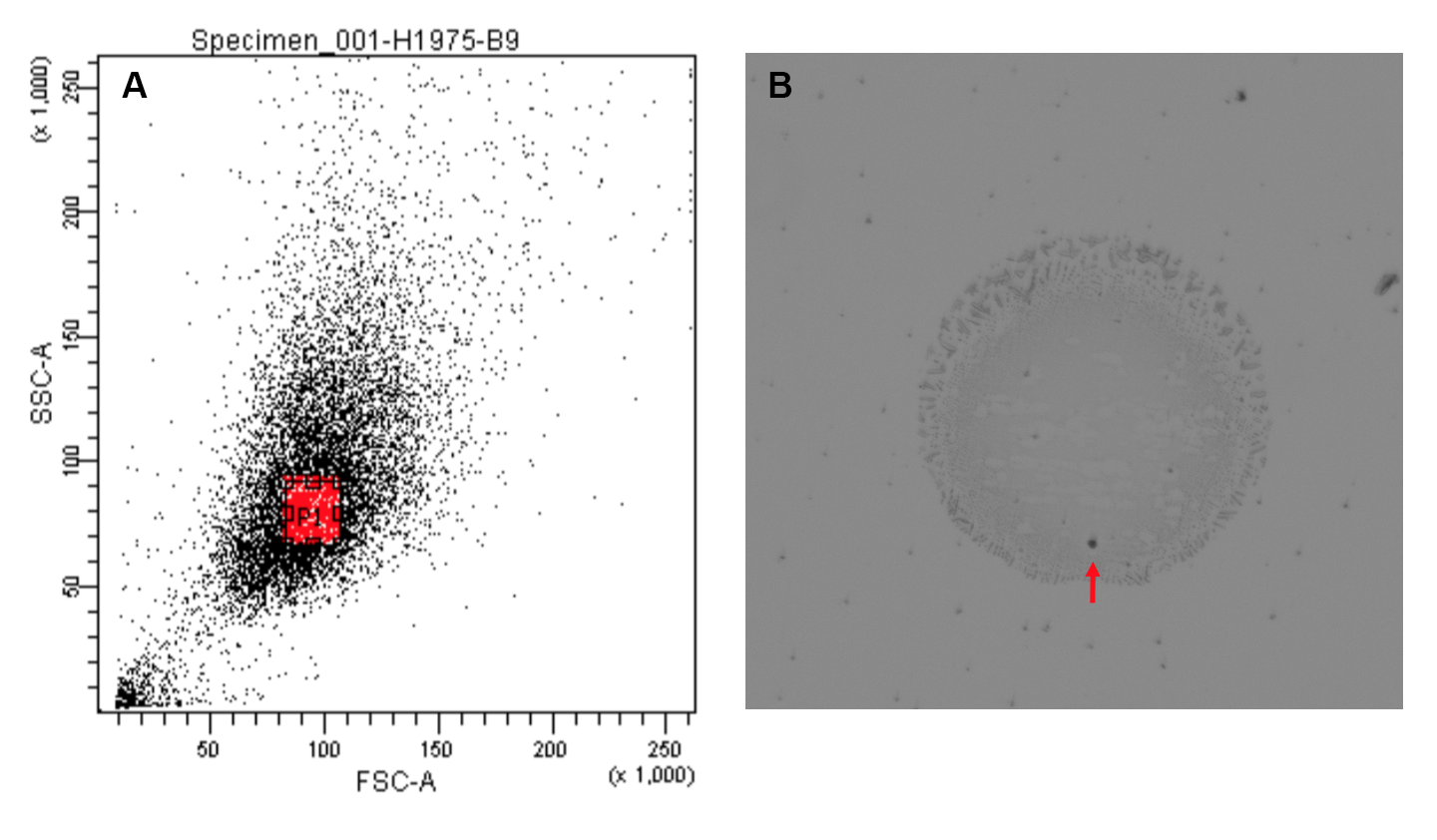

Supplement: Supplementary file 1 — Figure S1. Preliminary experiment for single cell isolation with flow cytometry (FCM). (A) Forward scattered light area (FSC-A) represents the cell size and side scattered light area (SSC-A) shows the number of cells. The red region represents the population of FCM collection. (B) H1975 cells in suspension were stained with Trypan blue and the single cells were sorted by FCM onto a microscope slide. The single cell (arrow) inside the droplet was observed under a microscope. (TIF 3317 kb) [file 12885_2019_5555_MOESM1_ESM.tif]
