# Supplementary material for: Intratumoral heterogeneity of EGFR-activating mutations in advanced NSCLC patients at the single-cell level
Source: BMC Cancer. 2019 Apr 23;19:369. doi: 10.1186/s12885-019-5555-y (PMC6480785; doi:10.1186/s12885-019-5555-y)
Supplement: Supplementary file 2 — Table S1. Clinicopathological features of six patients with non-small cell lung cancer. Table S2. Efficiencies of nested PCR. (DOCX 24 kb) [file 12885_2019_5555_MOESM2_ESM.docx]

**Table S1. Clinicopathological features of six patients with non-small cell lung cancer.**

| **Patient no.** | **Sex** | **Age** | **Performance**  **score** | **Smoking status** | **Stage** | **Tumor** | **Histology** | **EGFR**  **status** | **TKI**  **treatment** | **PFS**  **(month)** | **Response to TKIs** |
| --- | --- | --- | --- | --- | --- | --- | --- | --- | --- | --- | --- |
| No.1 | male | 87 | 1 | Smoker | IV | Lung | Ad | L858R | First-line | 19 | PR |
| No.2 | male | 47 | 2 | Non-smoker | IV | Lung | Ad | L858R | First-line | 22 | SD |
| No.3 | male | 42 | 2 | Smoker | IV | Lung | Ad | L858R | First-line | 15 | PR |
| No.4 | male | 76 | 1 | Smoker | IV | Lung | Ad | L858R | First-line | 5 | SD |
| No.5 | male | 60 | 2 | Non-smoker | IV | Lung | Ad | L858R | First-line | 3 | PD |
| No.6 | female | 64 | 2 | Non-smoker | IV | Lung | Ad | L858R | First-line | 1 | PD |

( Ad: adenocarcinoma; PD: progressive disease; PR: partial response; SD: stable disease.)

**Supplementary Table 2. Efficiencies of nested PCR**

| **Patient no.** | **tumor cells number** | **Nested PCR efficiency** | **P-value** |
| --- | --- | --- | --- |
| Long PFS group |  |  | 0.077 |
| No.1 | 23 | 19 (82.6%) |  |
| No.2 | 24 | 21 (87.5%) |  |
| No.3 | 23 | 19 (82.6%) |  |
| Total | 70 | 59 (84.3%) |  |
| Short PFS group |  |  |  |
| No.4 | 20 | 18 (90.0%) |  |
| No.5 | 23 | 17 (91.3%) |  |
| No.6 | 22 | 22 (100.0%) |  |
| Total | 65 | 61 (93.8%) |  |
